# Sex Differences of Lp(a) and Association With Mortality in a Primary Prevention Cohort

**DOI:** 10.1016/j.jacadv.2025.101596

**Published:** 2025-02-10

**Authors:** Tessa M. Zeis, Danielle Brennan, Madlaina Costa-Scharplatz, Leslie Cho

**Affiliations:** a Cardiovascular Medicine, Heart, Vascular Thoracic Institute Cleveland Clinic, Cleveland, Ohio, USA; b Novartis Pharma, Basel, Switzerland

**Keywords:** cardiovascular disease, cholesterol, lipoprotein (a)

**What is the clinical question being addressed?**

To investigate the sex differences in Lp(a) levels and associations with all-cause mortality.

**What is the main finding?**

A higher proportion of females had elevated Lp(a) levels than males, though overall improved survival compared to males.

Lipoprotein (a) [Lp(a)] is a genetic and causal risk factor for the development of atherosclerotic cardiovascular disease (ASCVD). The aim of this study was to investigate sex differences in clinical and laboratory characteristics of primary prevention patients with elevated Lp(a) and its associations with all-cause mortality by sex.

Patient data were obtained from the Preventive Cardiology Information System, a database of clinical and laboratory information on primary prevention patients seen at Cleveland Clinic Preventive Cardiology Clinic from 1999 to 2018. Patients were at least 18 years of age. Primary prevention was defined as having no history of coronary artery disease, coronary and peripheral arterial revascularization, nor atherosclerosis. Mortality data were gathered from the Social Security Death Index, the Ohio State Department of Health Vital Statistics, and electronic medical record. The Preventive Cardiology Information System database is approved by the Cleveland Clinic Institutional Review Board.

Primary outcome evaluated the Lp(a) distribution differences between sexes. Patients were grouped into Lp(a) categories (<30, 30-50, 50-70, 70-90, >90 mg/dL). Secondary outcomes, including all-cause mortality, were assessed using time to event analyses and age-adjusted Cox proportional hazards models. All statistical analyses were conducted using SAS version 9.4 (SAS Institute).

A total of 8,136 patients with Lp(a) results were included with 4,632 females (57%) and 3,504 males (43%). The number of female patients in each category were 2,545 (Lp(a) <30), 614 (30-<50), 437 (50-<70), 341 (70-<90), and 695 (>90). The mean age of females and males was 53.3 ± 13.4 vs 52.0 ± 13.5 years, respectively. There were more Black patients included in the female group than males (15.1% vs 8.1%). Females had higher rates than males of all comorbidities evaluated, particularly, hypertension (24% vs 17%), lipid abnormalities (36% vs 28.6%), history of psychiatric diseases (15% vs 5%), and rheumatoid diseases (11% vs 5.6%). Total cholesterol, low density lipoprotein, high density lipoprotein, and high sensitivity CRP levels were slightly higher in females, while triglyceride levels were higher in males. There was no difference in proportion of patients with diabetes between sexes. Nine percent of males and females were on statins at baseline. Proprotein convertase subtilisin/kexin type 9 inhibitor therapy data were not collected in this study.

There was a higher proportion of females than males in each Lp(a) category. The greatest difference in proportion was at severely elevated Lp(a) levels >90 mg/dL (68.5% females vs 31.5% males). Approximately 31.8% of all females had Lp(a) levels >50 mg/dL, while only 25.5% of all the males had Lp(a) levels >50 mg/dL.

Females had improved survival compared to males with comparable Lp(a) levels through 20 years of follow-up. There was no statistically significant difference in all-cause mortality in males when comparing varying Lp(a) levels (<50/≥50, <70/≥70, and <90/≥90 mg/dL). Although there was no difference in all-cause mortality among females when comparing Lp(a) levels at the lower cutoffs, there was a trend toward a difference in females with Lp(a) <90 and ≥90 mg/dL (HR: 1.32; 95% CI: 1.0-1.74) (Figure 1).

**Figure 1:**
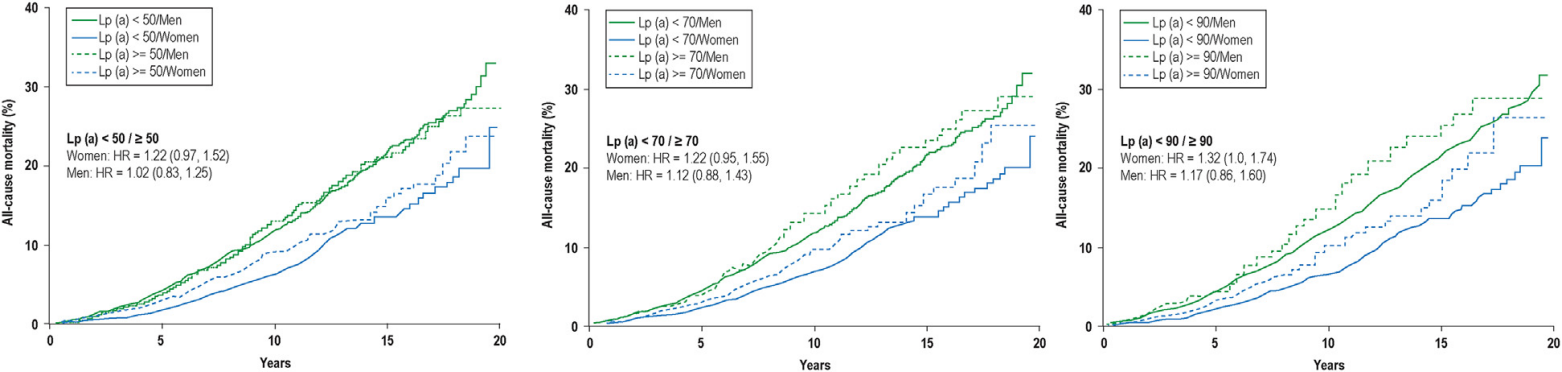
All-Cause Mortality for Females vs Males at Varying Lipoprotein (a) Levels

Overall, a higher proportion of females had elevated Lp(a) levels than males in our cohort. This difference was greatest at higher Lp(a) levels (>90 mg/dL). This phenomenon has been seen particularly in patients with ASCVD. Nissen et al^1^ found higher Lp(a) levels in females compared to males in a cohort of patients with ASCVD. Further studies have shown significantly higher Lp(a) levels in females than males only after age 50 and this effect was partially mitigated when on hormone replacement therapy, suggesting menopause could be contributing to increased Lp(a) levels.^2^ We did not specifically evaluate for menopause in our cohort.

Despite having overall higher Lp(a) levels and more comorbidities, females had improved survival compared to males; however, in females, there was a trend toward worsened mortality when Lp(a) was >90 mg/dL. Few studies have directly evaluated sex differences in Lp(a) and the relationship to mortality. While our study did not specifically evaluate CVD mortality, Markus et al^3^ found a significant increase in CVD mortality in women compared to men with type 2 DM which may be explained by elevated Lp(a) concentrations. This study and ours suggest that severely elevated Lp(a) levels may be a more important risk enhancer for mortality and ASCVD in females than males.

Overall, our findings suggest a sex difference in the distribution of Lp(a) and all-cause mortality. Especially at severely elevated levels, Lp(a) may be a more important predictor of mortality in females compared to males. This study is unique in that we were evaluating sex differences in Lp(a) in the primary prevention population. Further investigations in primary prevention population are warranted as targeted therapies to lower Lp(a) are developing.

## Funding support and author disclosures

This research was funded by Novartis Pharma AG. Dr Costa-Scharplatz is an employee of Novartis. All other authors have reported that they have no relationships relevant to the contents of this paper to disclose.

## References

[1] Nissen S.E., Wolski K., Cho L., Lp(a)HERITAGE Investigators (2022). Lipoprotein(a) levels in a global population with established atherosclerotic cardiovascular disease. Open Heart.

[2] Simony S.B., Mortensen M.B., Langsted A., Afzal S., Kamstrup P.R., Nordestgaard B.G. (2022). Sex differences of lipoprotein(a) levels and associated risk of morbidity and mortality by age: the Copenhagen General Population Study. Atherosclerosis.

[3] Markus M.R.P., Ittermann T., Schipf S. (2021). Association of sex-specific differences in lipoprotein (a) concentrations with cardiovascular mortality in individuals with T2DM. Cardiovasc Diabetol.

